# Resiliency Training in Indian Children: A Pilot Investigation of the Penn Resiliency Program

**DOI:** 10.3390/ijerph110404125

**Published:** 2014-04-15

**Authors:** Aruna Sankaranarayanan, Chandrika Cycil

**Affiliations:** 1 Director Prayatna 57, Prithvi Avenue, Chennai 600018, India; 2 Department of Computer Science, Brunel University Uxbridge, London UB8 3PH, UK; E-Mail: chandrika.cycil@brunel.ac.uk

**Keywords:** resiliency, depression, children, attributional style, adolescents, optimism, PRP, urban India

## Abstract

This paper examines the effectiveness of the Penn Resiliency Program (PRP) in an urban Indian setting. The PRP is a program to prevent depression in early adolescence and has proved successful in changing children’s attributional style of life events. While the program has been successful in preventing symptoms of depression in Western populations, the current study explored whether this program could be effective with an Indian sample. The aim of the current study was twofold; first, to study the attributional style of early adolescents in India and identify negative effects (if any) and second, to gain insights in using the PRP as a tool to change explanatory styles in Indian children. A total of 58 children participated in the study (Intervention group *n* = 29 and Control group *n* = 29). An Analysis of Covariance comparing post-test scores on Children’s Attributional Style Questionnaire (CASQ) while controlling for baseline scores indicated that children in the intervention group exhibited a significant reduction in pessimistic explanatory style and an increase in optimistic orientation compared to children in the control group. This indicates that the program was effective in changing negative attribution styles among upper-class Indian school children. Future work may look into the longer impact of the program as well as further considerations into adapting the program for a middle class population.

## 1. Introduction

Depression is a debilitating disease; hence, preventing its onset is beneficial at the individual, familial and societal levels. In India, a growing population, poor levels of literacy in rural areas, varying social support amongst a myriad of other cultural factors are observed to be key predictors for rising cases of depression [1]. In the Southern city of Chennai alone (where the current study is situated), the prevalence of depression in a sample of middle aged men and women is estimated at around 15% [2]. These studies also found a disproportionately higher incidence of depression in women than men. In part, the discriminatory treatment of women accounts for many social and emotional problems in low-income countries [3,4]. Reviews of epidemiological studies indicate comparatively lower incidence of mental health problem in low income countries like India attributing this to protective factors associated with family, cultural and religious values [3,5]. However, there are concerns around the reliability of this data as methodological difficulties in adapting measurement tools [3] and gross underreporting of suicidal attempts, abuse and gender violence (proven to be strong predictors of anxiety and mood disorders) exists [6]. Further, adolescence is marked by significant physiological changes as well as an interaction of psychosocial influences from the child’s home, school and social environment.

Adolescents in India are reported to be at risk for a number of behavioural and emotional problems [7]. The recent rises in the number of school-based intervention programs, government-mandated posts of school counsellors, are all indicators of the need to support the psychological and emotional needs of school-age children. Several risk factors have been associated with poor mental health in adolescents including stressful family environments, coercive sexual encounters, gender discrimination and poor social support [4]. Expectedly, the presence of mental disorders such as anxiety, emotional and mood disturbances affect wellbeing by increasing the risk of suicide attempts in adolescents [6]. Adolescent girls are particularly more vulnerable to depression [8] than their male counterparts as they experience more parental pressures and restrictions with regard to lifestyle choices. Therefore protective factors of social and familial ties seem to contradict with gender discriminatory attitudes rooted in traditional Indian culture with regard to adolescent girls. The sheer magnitude of the population and lack of resources, the cultural factors such as family obligations, poor literacy and taboos associated with discussing mental health problems places an enormous task on mental health services [7].

Research on child and adolescent mental health in India has been brought to focus in the last decade and the aforementioned studies report on the epidemiology and associated risk factors of depression. However, managing psychological problems in India are complicated by the cultural and social associations with seeking help [9]. Given the complex issues surrounding this, previous research indicates, the need for community-based health promotion programs with generic goals [3] to target awareness on gender discrimination, infant mortality as well as mental health-where there is a current dearth of literature. In their evaluation of mental health services in low income countries [3] (including India) the authors discuss that the key to providing these services is to focus on capacity building efforts such as empowerment of women and strengthening adolescents and their families. Similar approaches to this have been previously suggested for adolescents in the form of integrated “adolescent health services” targeting educational, psychosocial as well as emotional and physical needs of adolescents within schools [10]. South India is very much rooted in traditional culture like other parts of India where the diagnosis of a mental disorder or seeking help and treatment is influenced by fear of discord in family and social relations. Hence, the inoculant idea of preventing poor health or promoting good health and well-being appears desirable [9].

### 1.1. Depression, Resiliency and the Penn Resiliency Program

Learned helplessness is a state wherein persistent negative events in an individual’s life leads him/her to experience a loss of control over the consequence and expectancy of negative events [11]. This has been associated with the onset of depression-arising from a condition of hopelessness [11,12]. According to the attributional theory of learned helplessness [11], depressive thoughts and depression are a result of persistent negative attributions to life events. Three attributional dimensions namely, internal-external, stable-unstable and global-specific were identified in explaining an individual’s susceptibility to depression. According to this explanation, individuals who explain bad events to internal, stable and global causes are said to have a negative or pessimistic explanatory style [12]. Pessimistic style of thinking has been associated with depressive symptoms and a pre-disposition for depression [13,14]. We note here that the terms “attributional style” and “explanatory style” has been used to refer to the same mechanism [15]. From this point in the paper, we will use the term “explanatory style”.

Studies have found that children at risk for depression (and those showing depressive symptomology) in contrast to their peers make more negative attributions, and tend to attribute negative events to personal and permanent causes and positive events to external and temporary ones [15,16,17]. Resiliency is the process of coping with adversity or adverse consequences of negative events [18]. Specifically, individual “protective factors” or specific capabilities are associated with coping flexibly with adversity [19]. Individuals thus use a repertoire of skills (drawing on past experiences and environmental factors) to face the challenges of a given situation. When they are persistently unable to cope with a situation, they are faced with a sense of “helplessness” and become vulnerable to depression [20].

The Penn Resiliency Program (PRP) is an intervention targeted at reducing risk factors for depression and promote resiliency is early adolescence [20,21]. It was developed to prevent depression in children and adolescents and has proved successful in changing children’s pessimistic explanatory style [22]. When appraising their performance, children with a pessimistic explanatory style have distorted perceptions and tend to pay more attention to negative features of events [23,24,25]. Cognitive behaviour therapy interventions involve correcting distorted perceptions by analysing self-talk and teaching adaptive skills like social problem-solving [26]. The PRP consists of two components-the cognitive and the problem-solving [21]. The first teaches children to identify negative beliefs and examine the validity of those beliefs and helps children develop cognitive flexibility when confronted with negative thoughts. In the second part of the program, children are taught to resolve interpersonal conflicts, communicate assertively and avoid the extremes of aggression and passivity. The program is administered through a series of role-plays, cartoons, games and homework assignments that teach and reinforce these concepts [20].

There is currently no literature linking explanatory style and depression in Indian children. However, some insightful cross-cultural findings exist. In studying the cultural influence on explanatory styles in undergraduates [27], Chinese students showed a more pessimistic style of responding to events in comparison to White Americans and Chinese-Americans. They tended to attribute positive events to external causes and negative events to internal causes. Some of these findings were related to socio-cultural values held by specific groups (e.g., being modest or self-effacing) as well as coming from a individualist or collectivist culture. Further, when the PRP was adapted for children in China [28], researchers found it to be successful in reducing symptoms of depression in children as well as predicting (and reducing) the risk of pessimistic explanatory styles in vulnerable (at-risk) children. However, some parts had to adapted to suit the cultural context; for example, assertiveness was not encouraged in Chinese culture. We noticed similar concerns in our study with an Indian school sample given that Indian culture is also collectivist.

### 1.2. Rationale and Need for Prevention

Interventions to tackle mental health tend to be underreported in the literature with respect to upper class Indian children even though the prevalence of mental health problems is said to be comparatively higher in upper and middle class Indian families [29]. The World Health Organisation’s (WHO) life-skills training program [30] is among the few recognised programs that aim at improving self-esteem, assertiveness and social skills in schools through group sessions. These have been popularised in rural areas and lower income schools in urban areas-though largely unreported. Recent changes in lifestyle choices and Westernisation indicate that adolescents in urban Indian cities face issues very similar to a Western population. In Goa [31], researchers found that adolescents are increasingly at conflict with traditional family value systems through modern lifestyle choices, such as dating and partying with friends. Furthermore private education in India is largely in English-making the idea of using an existing prevention program from a Western population feasible. One of the motivating factors for our study is that children from upper class Indian families have the exposure and English skills comparable with children in developed countries. With this knowledge, we were confident that children would be able to relate to aspects of the PRP in its original format before adapting it, like its implementation in China [28]. Using an established program would give us an indicator of what issues arise when adapting a program to a specific cultural context.

The aim of the current study was twofold-first, to study the explanatory style of early adolescents in India and identify any negative effects (if any) and second, to gain insights in using the PRP (imported from the U.S.) as a tool to change explanatory style in an Urban, upper-class Indian setting. While the program has been successful in both preventing and reducing symptoms of depression in Western populations [22,32], the current study examined if this program would be effective as a preventive tool in a cross-cultural sample. As the United States (where the PRP was originally developed) and India differ on a number of social, cultural, economic, and demographic dimensions, the relevance and feasibility of importing the PRP in its original format for an Indian population was considered. However, before adapting the program for Indian students, a necessary first-step would be to administer the program in its original format. We also discuss what aspects of the program worked and provide insights for future preventive programs.

## 2. Method

### 2.1. Procedure

The researchers sought approval to conduct the study directly from the Principal and Headmistress of the Primary School in consultation with the school counselor. Prior to the study’s commencement, parents of both groups were informed about the study by the Headmistress during a Parent-Teacher meeting at school. There were no objections raised to the study and consent was obtained. Next, participating children were informed about the study by their class teachers. The PRP lessons were held twice a week over Term 3 (January–March) of the School Year during school hours on the school premises. We chose to work during school hours to avoid children returning home late as well as missing afternoon extracurricular activities. A typical school day is broken down into 45 min blocks with a half hour snack break in the middle of the day. Two blocks of 45 min each were used to conduct the PRP.

The intervention group was further divided into two sub-groups with 15 children in one group and 14 in the other. Each sub-group was led by one of the researchers. The principal researcher holds a doctorate in Developmental Psychology and the co-researcher had a Master’s in Psychology at the time. The intervention groups received 22 h of instruction in the PRP over three months. The attendance in each intervention sub group was 95% and 96%. The researchers studied the PRP program manual closely and reviewed each lesson in great detail together. They had copies of the lesson and homework for each session and practiced by rehearsing the first two lessons in advance of meeting with the students. The leaders also met after each lesson to discuss how each activity was received by the students. These steps were taken to ensure that the group leaders adhered closely to the original program and to minimize differences that may arise due to the groups being led by different leaders.

### 2.2. Sample

The sample comprised of 58 children studying in two sections of Grade V in a relatively affluent school in Chennai, India. Since its development, the PRP targeted preventing depression in early adolescents (10–14 years) [21]. The aim is to equip children with psychosocial skills that would help them cope with the impending physical, social and emotional changes in adolescence [20]. In this school, Grades II to V are regarded as Junior School. In every Indian school, grades are divided into manageable groups called “sections” such as “section A”, “section B” and so on. Typically there is no rationale for assigning a child to a section-they are randomly assigned to sections and are representative of a range of academic abilities. The classification of children into sections is primarily to maintain gender and student-teacher ratios. This particular school had only two sections, A and B, one served as the control and the latter as the intervention group. English was the medium of instruction in the school. Refer to Table 1 for details of the sample.

In Urban India, middle- and upper-class children tend to attend mainly private schools. Private institutions use different curricula but the most widely used and well-recognised are the CBSE (Central Bureau of Secondary Education), ICSE (Indian Council for Secondary Education) and State-based boards (varies by each State). The school selected for the program followed the ICSE curriculum—a more academically challenging program than ones offered by the State-based Indian schools. The school catered to children from higher-socioeconomic families and hence children were likely to be exposed to the Western culture and be able to relate to the original material of the PRP. Children from the selected school are likely to have imbibed aspects of Western culture from a number of sources—watching Western programs on television, Hollywood movies, Disney cartoons, travelling overseas and have family living and visiting from other parts of the world. The impact of students’ family background is elaborated on in the Discussion section.

**Table 1:** Description of sample.

Group	Gender		Mean Age (in years)
	Boys	Girls	
Intervention	*n =* 17	*n =* 12	9.5
Control	*n =* 17	*n =* 12	9.6

### 2.3. Measures

Prior to the intervention, all 58 children were administered the Children’s Attributional Style Questionnaire (CASQ) [33]. The 48-item questionnaire developed by Kaslow *et al.* [33] comprises of multiple-choice items and yields scores for a child’s explanatory style for good (24 items) and bad (24 items) life events along three sub scale dimensions-permanent *vs.* temporary, pervasive *vs.* specific, and personal *vs.* other. Composite scores of “total good” (labelled TG) and “total bad” (labelled TB) are obtained by summing each of the three individual sub scale dimensions for bad and good events respectively. Categorical descriptions of “optimistic-almost invulnerable to depression”, “average-somewhat depressive” and “pessimistic-at marked risk for depression” are also assigned to children (based on a range of scores) based on norms for boys and girls separately. In a study of 96 elementary school children in the U.S. [13] reliability of the CASQ over a six month follow-up for the composite sub-scales was consistent with Cronbach alpha co-efficients of 0.71 and 0.66 for total good and total bad respectively [13]. Children who attribute negative events to temporary, specific, and external factors are classified as optimistic. Likewise, those who attribute positive events to permanent, pervasive and personal factors are also classified as optimistic. Pessimistic children tend to attribute positive events to temporary, external factors and when faced with negative events, they tend to engage in self-blame and generalise setbacks.

The rationale behind using the CASQ as opposed to other instruments was because we did not carry out a screening study with an intention to treat depressive symptoms in vulnerable children. The aims of the study were to identify and change negative explanatory styles in children and to pilot test the PRP as a preventive program in Indian children. In this sense, we used the program from the view of “prevention” *i.e.*, to provide intervention where the disorder was not identified thereby reducing the risk associated with depression [20]. We were in fact surprised to note that many children in Grade V were showing signs of strong negativity in responding to events. This is consistent with the underlying assumption of the development of the PRP wherein pessimistic thinking can develop as early as pre-adolescence between 10 and 12 years [20,22]. The questionnaire was read aloud to children by the researchers, item by item, and children circled their responses. This procedure was followed preceding intervention and after the program. A week after the last PRP session, both sections comprising a total of 58 children were again administered the CASQ again. Scoring of the scales was done by the co-researcher and statistical analysis was done using SPSS v20.

### 2.4. Statistical Methods

To measure the effect of the intervention (PRP) on explanatory style, we used ANCOVA controlling for baseline scores across control and intervention groups. The pre-test TB and TG scores were used as covariates. At baseline, the children in control and intervention groups did not show significant differences in their composite TG and TB scores.

## 3. Results

**Figure 1:**
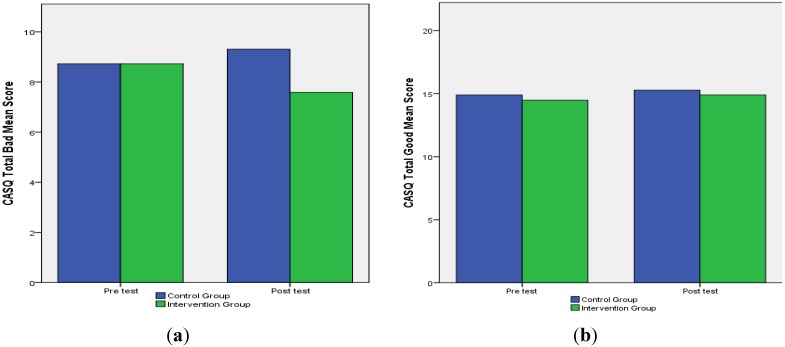
(**a**) Mean TB scores; (**b**) Mean TG scores.

Our main goal was to see whether the PRP would have an effect on changing the pessimistic explanatory style of children in the intervention group. The composite TB and TG scores on the CASQ indicate whether a child’s explanatory style is optimistic or pessimistic. Therefore, a higher TB score indicates a pessimistic explanatory style and a higher TG score indicates an optimistic explanatory style. In Figure 1a we observe that following the PRP, the intervention group showed a distinct lowering in their mean score of negative events (TB score), while the control group showed an increase. In Total Good events, we notice only a slight increase in both intervention and control groups.

**Figure 2:**
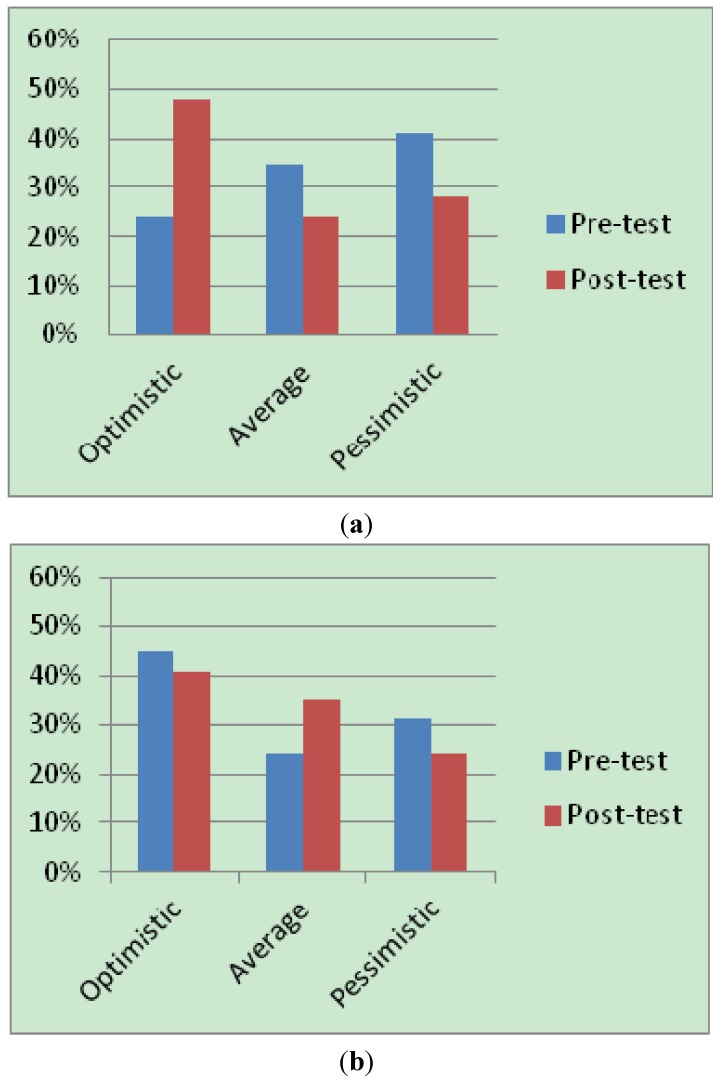
(**a**) Percentages based on TB scores; (**b**) Percentages based on TG scores.

Children’s scores on the composite good and bad scores also provide categorical descriptions of explanatory style and their vulnerability to depression (refer Section 2.3). A pre and post-test comparison of children in the intervention group (see Figure 2a,b) indicated that a noticeable percentage showed a reduced pessimistic explanatory style. On descriptive analysis, comparison of their scores on the TB sub-scale dimension, we observed a doubling in the number of children who were classified as “optimistic” or “invulnerable to depression” (7 at pre-test and 14 at posttest). For the TG score, we did not notice such a significant difference. However, the post-test scores indicate that the direction of scores were positive (*i.e*., a lowering of children classified as pessimistic).

An ANCOVA predicting explanatory style of negative effects (CASQ, Total Bad) revealed a significant effect of condition, *F*(1, 57) = 6.10, *p* < 0.05 (*R^2^* = 0.32). The two groups did not differ in their positive explanatory style (see Table 2). Therefore, we see that following the intervention, children in the intervention group experienced a lowering in their TB score. In their experience of good events (TG) children in the program show a small increase in their mean score.

**Table 2:** Differences across intervention and control groups on dimensions of the CASQ.

Dimensions(CASQ ^b^)	Condition	Intervention *N* = 29		Control *N* = 29		Statistic ^a^
		Mean	SD	Mean	SD	
Total Bad (TB)	Pre-test	8.72	2.17	8.72	2.88	*F*(1, 57) = 6.10 *
	Post-test	7.59	3.08	9.31	2.99	
Total Good (TG)	Pre-test	14.48	3.10	14.90	2.76	*F*(1, 57) = 0.067
	Post-test	14.90	3.28	15.28	2.81	

Notes: **a** Analyses of covariance were performed on CASQ post test scores on completion of PRP on both intervention and control groups. Pre-test scores were used as covariates across total good and bad dimensions. **b** CASQ is the Children's Attributional Style Questionnaire. * *p* < 0.05.

Previous work indicates that at pre-adolescence, boys tend to have a more pessimistic explanatory style than girls [28]. However, we found that girls in our study had slightly higher TB scores than the boys (see Table 3). We infer that this is may be due to cultural differences in Indian children-gender differences in expectations from girls are reinforced quite early. However, girls (at pre-intervention) started with a higher TG score, which reduced at post intervention. This shows that the explanatory style did not remain consistent over measurement periods. One explanation for this could be because children’s responses to life events are still taking shape and they may be inconsistent in their appraisal of events. Further follow up as children move into middle adolescence is required to see whether these styles sustain or change over time.

**Table 3:** Differences across gender in the intervention group on dimensions of the CASQ.

Dimensions(CASQ )	Condition	Gender	Mean	SD	*t* ^a^	*p*-Value ^b^
Total Bad (TB)	Pre-test	Boys (*n* = 17)	8.29	2.49	−1.29	NS *
		Girls (*n* = 12)	9.33	1.49		
	Post-test	Boys (*n* = 17)	7.53	3.02	0.12	NS *
		Girls (*n* = 12)	7.67	3.28		
Total Good (TG)	Pre-test	Boys (*n* = 17)	13.94	3.15	−1.13	NS *
		Girls (*n* = 12)	15.25	2.98		
	Post-test	Boys (*n* = 17)	15.18	3.66	0.54	NS *
		Girls *(n* = 12)	14.50	2.74		

Notes: **a** Tests of difference were performed to study effects of intervention across gender. **b** Levels of significance. * Not significant.

## 4. Discussion

Despite cultural differences, the PRP was effective in reducing pessimistic thinking and promoting an optimistic explanatory style among school children in India. It is difficult to interpret explanatory style as the validity of the CASQ as a predictor of depression has not been established. There are also cross-cultural differences in attribution styles not previously explored in the Indian population. For example, Americans are more likely to attribute success to inherent abilities and failure to external factors while Japanese and Chinese tend to do the reverse [27,34]. In our observation, Indian students reflect similar patterns of attribution as their Asian counterparts. In our sessions, children were less likely to attribute negative events to external factors. This is something that is culturally encouraged in Indian culture as children are taught to avoid confrontation and externalising blame. Another aspect that warrants discussion is the higher TB score of pre-adolescent girls in our study. We notice this finding may be an important cultural feature of negative explanatory style in Indian girls. In the Indian population, girls cope with distinctive stressful factors like harmonizing academic pursuits along with parental expectations and early marriage. In many cases, the transition into adulthood for Indian girls may happen earlier; therefore, the need for psychosocial support may be required earlier on as well.

One aspect that seemed culturally unfamiliar to our sample was assertiveness. Children tended to think that being passive was more appropriate than being assertive and “getting your way”. In fact, some children equated being assertive with being aggressive. However, with sufficient modeling and explanation of how assertiveness was different from aggressiveness, children were able to exhibit assertiveness. It is important to note here that assertiveness is not a trait that is explicitly taught to Indian children. However, studies show passiveness in Indian adolescents is associated with increasing susceptibility to problem behaviours like substance abuse, behavioural misconduct and peer pressure, [4,6,31]. This has led mental health professionals and guidance counsellors in India place more emphasis on including assertiveness training as a part of life skills training and psychosocial intervention programs. However, assertiveness is not encouraged with elders, parents and teachers and any further work on this may need to clearly specify to Indian children where it may be applicable. In other Asian cultures like China where the PRP was adapted, they similarly found that assertiveness needed to be toned down to the cultural context [28]. Further, children’s conflicts with traditional Indian family values (including valuing elders’ opinion over their own) seem to create stressful home environments that predispose children to anxiety and other mental health issues [10,31].

Long-term follow-up at specific intervals may be required to see if any of these children are at risk or have developed depressive symptoms. While we did not see any significant differences in the intervention group with respect to gender, Indian adolescents (particularly girls) are at risk for psychological problems from stressors like academic pressures, pressure from peers and parental expectations as they progress into adolescence. This is supported with data from India show that depression increases with age in adolescence where incidence of depressive symptoms sees a steep increase in middle to late adolescence [35,36]. Further follow-up may also help in observing whether explanatory styles remain fairly consistent over time. A caveat has to be noted. Children in the control group did not receive a placebo intervention. Thus, the extra adult attention of two outsiders engaging the children in the intervention group may have contributed to the result.

It should be noted that children in this sample were from relatively affluent backgrounds—in this school, several children were admitted after their families returned from spending considerable time abroad and hence were likely to be exposed to aspects of the Western culture. Further, non-traditional lifestyles of adolescents are fairly common in major Indian cities. In this light, the concerns and issues of children in this school were similar to those of Western populations. Issues such as bullying; interactions with the opposite sex, overcoming peer pressure were familiar with the children in this school. Given this context, we saw the assertiveness aspect as an essential part to include while adapting the program to an urban Indian population. Similar concern to address psychosocial issues of adolescents were found in other Indian cities like Goa and Chandigarh [7]. Hence prevention programs in urban Indian cities may call for a different cultural adaptation as compared to other Indian populations. Whether this program will be effective in a school catering to children from middle-class backgrounds in India needs to be studied.

Another feature of the program noted by the researchers was that the language and vocabulary was above average for an Indian state school curriculum. In India, very few state-based school curricula cater to such a high level of English proficiency. The current sample showed no difficultly comprehending various aspects of the program. One reason for this is that the school curriculum was at a higher level than state board schools. It is difficult to generalise the linguistic and cultural appropriateness of the program to the general Indian population as the sample chosen to participate was a very selective group. Students of other school curricula may require a more simplified version of the lessons in order to fully comprehend and internalise them. Hence, a program for urban middle class or rural school populations may warrant significant modifications.

Other qualitative observations were notably observed with regards to the implementation of the program in India. While it is not possible to provide detailed descriptions of these qualitative findings, we provide some key insights that may encourage future work with similar populations. Our experience in the sessions reflected that the program might work better in smaller groups. While the researchers worked with 15 children in each intervention group, we felt the ideal set would be around 6–8. This was because we found that children did not always get a chance to speak up and the quiet ones got left out. With regard to the program structure, the children seemed to prefer some activities over others. For example, the completion of homework lessons was inconsistent with some children giving them in regularly and others occasionally. In general, homework within the Indian context, is perceived quite negatively, hence future work on such interventions may choose to avoid too much written assignments for homework. We also found that some ice breakers or introductory activities may be a useful addition to the program. This may serve as a gentle introduction to the program and the facilitators.

## 5. Conclusions and Future Considerations

Overall, this study found that the Penn Resiliency Program developed for Western children was effective in changing the negative explanatory style of upper-middle class Indian children. However, further research is required to see if the results of this study generalise to other segments of the Indian population. Largely, we found that the children were very enthusiastic and responsive. Despite the researchers being outsiders, the children were able to warm up to them half way through the program. Some female students spoke about problems relating to their families and siblings and shared how the program homework was helping them. While boys did not take this initiative, in both groups, they participated actively in all aspects of the program. Similar results were found in other implementations of the PRP [32] indicating that the program meets the needs of pre-adolescent girls. This is indeed a promising finding as the scope of PRP or an adaptation to be applied in all-girls setting in India (given the unique cultural context) is certainly crucial and may be explored in future work.

Furthermore, the longer term impact of the intervention may be studied through longitudinal follow-ups. Previous follow up studies indicate sustained optimistic attitudes and increasing optimistic outlook in children who underwent training in the PRP as compared to their peers who did not receive training [2]. Another aspect that we did not consider for this pilot and which may have potential for future work in India is a Parent Training Program. Other investigations of PRP discuss the usefulness of including parent and teacher involvement in the program [20,37]. This may be a component that is further explored in the Indian setting. The current research and prior work seems to indicate that problems in Indian adolescents may be aggravated from strained relationships with family members-siblings, parents and child-rearing practices [7]. In fact, while conducting the study, a number of examples in the homework exercises indicated that children had conflicts with siblings and parents. However, several protective factors of family support has also proved to be a deterrent to developing mental health problems [5,31], which could encourage future work in identifying and nurturing these factors and discouraging factors that cause dissonance in parent-child relationships.

In addition, the validity of the CASQ as a predictor of depression has not been studied in the Indian context. Differences in explanatory style of girls and boys may be further studied on follow up to see if these styles remain consistent or fluctuate during adolescence. Further application of the PRP with larger groups of children and across more schools may also be pursued. Thus, further studies of attribution styles as predictors of depression in the Indian context are required. Moreover, indigenous programs and tools of assessment may be developed that factor in local cultural beliefs, patterns and preferences where the program cannot be applied in its original format.
